# Deep Splicing Code: Classifying Alternative Splicing Events Using Deep Learning

**DOI:** 10.3390/genes10080587

**Published:** 2019-08-01

**Authors:** Zakaria Louadi, Mhaned Oubounyt, Hilal Tayara, Kil To Chong

**Affiliations:** 1 Department of Electronics and Information Engineering, Chonbuk National University, Jeonju 54896, Korea; 2 Advanced Electronics and Information Research Center, Chonbuk National University, Jeonju 54896, Korea

**Keywords:** alternative splicing, computational biology, deep learning, splicing code, splicing predictor

## Abstract

Alternative splicing (AS) is the process of combining different parts of the pre-mRNA to produce diverse transcripts and eventually different protein products from a single gene. In computational biology field, researchers try to understand AS behavior and regulation using computational models known as “Splicing Codes”. The final goal of these algorithms is to make an in-silico prediction of AS outcome from genomic sequence. Here, we develop a deep learning approach, called Deep Splicing Code (DSC), for categorizing the well-studied classes of AS namely alternatively skipped exons, alternative 5’ss, alternative 3’ss, and constitutively spliced exons based only on the sequence of the exon junctions. The proposed approach significantly improves the prediction and the obtained results reveal that constitutive exons have distinguishable local characteristics from alternatively spliced exons. Using the motif visualization technique, we show that the trained models learned to search for competitive alternative splice sites as well as motifs of important splicing factors with high precision. Thus, the proposed approach greatly expands the opportunities to improve alternative splicing modeling. In addition, a web-server for AS events prediction has been developed based on the proposed method.

## 1. Introduction

Alternative splicing is the key contributor to human transcriptome diversity by producing multiple messenger RNA (mRNA) from a single pre-mRNA. In this process, the non-coding intronic sequences are removed and exons are connected in different combinations. It is known that around 95% of the human genes with multiple exons are alternatively spliced [1]. The most common AS events are skipped exons (cassette exons), retained introns, and exons with an alternative 3’ or 5’ splice-site selection [1,2]. The regulation of this mechanism is highly variable and complex. Furthermore, genomic variation at the level of the sequence of the transcript can cause defects in splicing. This misregulation contributes significantly to human disease [3,4] and multiple types of cancer [5]. Recently, researchers built computational models, known as ‘splicing codes’, to predict AS outcome from DNA sequences alone and independently of existing studies, such as disease annotation and population data [6]. Such models were successfully applied to predict splicing-altering disease mutations [6]. Thus, it is believed that they can guide us to a better understanding of the mechanisms involved in splicing to develop personalized medicines.

Two major types of splicing code have been introduced: models for predicting the inclusion frequency of an exon [7,8,9] and models for distinguishing between constitutive exons and alternatively spliced exons [10]. For predicting the exact inclusion level of an alternatively spliced exon, factors from both the donor and the acceptor were shown to be necessary [11]. On the other hand, for the task of distinguishing between the constitutive and spliced exons, features from the local sequences around the exon junction itself have been studied in literature and reported to be the most critical [10,12,13,14]; Constitutive exons are known to have distinguishable characteristics from alternatively spliced exons such as their splice-sites strengths [12,14], the exon/intron architecture [15] and phylogenetic conservation around the exon junction [16,17]. The correlation of these factors with alternative splicing has been further examined exclusively and combined. For instance, Shepard et al. [12] studied the internal exons recognition based on the combined strength of the flanking 3’ and 5’ splice sites. Busch and Hertel [10] took a step further and presented an analysis of 262 unique features extracted from the exon and the two neighboring introns sequences to distinguish between the constitutive exons, cassette exons, and exons with alternative 5’ or 3${}^{\prime }$ splice sites. Their model efficiently identifies internal exon type for rarely and moderate occurred alternatively spliced events. However, for the frequently occurring AS events, such as highly included exons that have similar behavior to constitutive exons, the performance breaks down to around 0.62 in terms of area under the receiver operating characteristic curve (AUC) and to only 0.56 for high included cassette exons class. These studies have shown that none of the available RNA features could differentiate between constitutively spliced exons and high included alternative exons.

In parallel, the use of deep neural networks (DNN) for the automatic extraction of features rather than handcrafted construction has improved the state-of-the-art performance for many genomics tasks [18,19], such as predicting transcription factor binding site [20,21], Branch point selection [22,23], RNA protein-coding [24], DNA methylation [25]. The most successful variant of DNN is convolution neural networks (CNN), which have been recently used for mapping the data through multiple layers, where deeper layers represent a higher level of abstraction. Such techniques are proved to be more practical and simpler than constructing features by hand that can be very laborious and complicated.

In the field of AS modeling, Zhang et al. [26] proposed a classifier based on CNN for splice junctions derived from primary RNA-seq data. The biggest progress in the field of AS was in predicting splice sites such as SpliceRover [27] and SpliceAI [28] that outperformed the previous methods for identifying splice donor and splice acceptor sites using genomics sequences. Furthermore, Bretschneider et al. developed COSSMO [29] a deep learning approach for modeling the competition between splice sites. However, none of these models is designed to differentiate between different types of AS events such as distinguishing constitutive exons from the skipped ones. We, therefore, believe that there is still a significant room for improvement and analysis for the task of classification of AS events.

From this review, two main questions are raised: can alternatively spliced exons with high inclusion/usage levels be distinguished from constitutive exons using their local characteristics? More generally, can deep learning improve the accuracy compared to the features presented previously?

To answer these questions, we present a deep learning approach for classifying a human internal exon according to its alternative splicing behavior using its immediate raw RNA sequence. We train four models for different classification tasks. The architectures of our models are based on CNN where the kernels are trained in a supervised way and can be considered similar to position weight matrices (PWMs). Also, we analyze their performance across various AS frequencies and compare with the state-of-the-art method. Finally, we have developed a user-friendly web-server for predicting AS events and made it freely available at https://home.jbnu.ac.kr/NSCL/dsc.htm.

## 2. Materials and Methods

In this section, we introduce dataset preparation, the proposed models, and implementation details.

### 2.1. Dataset Preparation

To create the dataset used for training and testing the splicing code that classifies human internal exons, we follow a similar procedure to Busch and Hertel [10]. We extract a full list of splicing events from the latest update of the HEXEvent database [30]. HEXEvent is a freely available database that compiles a genome-wide exon data sets based on known isoforms from the UCSC Genome Browser (GRCh38/hg38) [31] as well as expressed sequences tag (EST) information. A splicing event in this context is a human internal exon characterized by the inclusion level (inclLevel) and the alternative 3’ and 5’ splice-sites usage levels (3usageLevel and 5usageLevel) as defined by Busch and Hertel [30]. For this work, we focus on studying splicing behavior and regulation generally but not tissue-specific splicing. Therefore, our original data is derived using ESTs from various tissues [30]. In total, we create four sets of events:

- A set of exon skipping (ES) or cassette exon events: exons that can be either excluded or included with a frequency value known as inclLevel (inclLevel ∈ [0, 1]) and do not have any other alternative splice site.
- A set of exons having an alternative 3’ (ALT3) splice site used with a frequency value known as 3usageLevel (3usageLevel ∈ [0, 1]).
- A set of exons having an alternative 5’ (ALT5) splice site used with a frequency value known as 5usageLevel (5usageLevel ∈ [0, 1]).
- A set of constitutive exons (CON): internal exons with no ESTs evidence of any type of alternative splicing; the inclusion level and all the splice sites usage levels are equal to 1.

**Figure 1:**
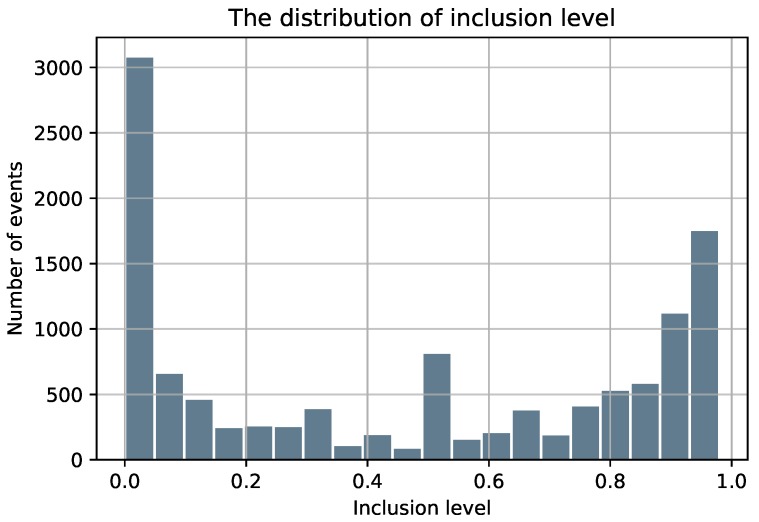
The distribution of the inclusion level of the skipped exons.

It is well-known that sequencing and alignment exhibit many biases. Consequently, to maximize the quality of the splicing code and avoid noisy training data, all exons in the dataset are a result of strict filtering to ensure a sufficient supporting number of ESTs for all alternative splicing cases (inclusion/exclusion). A skipped exon is included if it is supported by at least 20 ESTs and an alternative splice site should be supported by at least 4 ESTs. For the alternative exons (ES/ALT3/ALT5), we only keep the ones that display a single type of AS. For instance, the exons that have both alternative 3’ and 5’ splice sites were not included in our dataset. Finally, a minimal length of 25nt for the exons and a length of 80nt for the neighboring introns are required as exons/introns shorter than 25nt/80nt are usually caused by sequencing errors and they represent less than 1% of the exon and intron length distributions [32]. As shown in Figure 1, the majority of AS events are biased toward high included (>90%) and low included (<10%). Table 1 summarizes the final obtained dataset.

### 2.2. Features Selection

**Figure 2:**
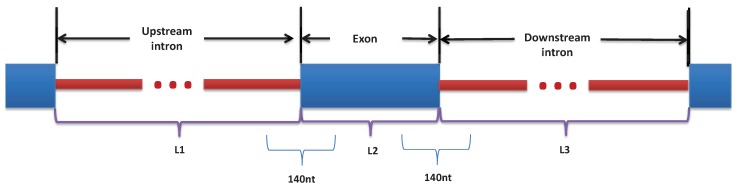
Illustration of the features selection from ±70nt around the exon/intron junction, L1: the length of the upstream intron, L2: the length of the exon, and L3: the length of the downstream intron.

For each event, we extract RNA sequences from ±70 nt around the exon/intron junctions, to get a total of 2 × 140 nt for every exon in the dataset. The motivation of using 70 nt is based on previous studies [33] and our initial experiments. To represent the four possible nucleotides, each of the 140nt sequences is one-hot encoded into a 140 × 4 binary matrix. In addition to the sequence features, several studies have shown that exon/intron architecture can influence splice-site recognition [15,34]. Therefore, we use three features representing the length of the exon (L2) and the length of its neighboring introns (L1 and L3) that are obtained by aligning the exon position to the Human Reference Sequence (GRCh38/hg38) and normalized by the mean and standard deviation of the lengths of the internal exons and the lengths of the introns in the human genome. Figure 2 illustrates feature selection.

### 2.3. The Proposed Models

**Figure 3:**
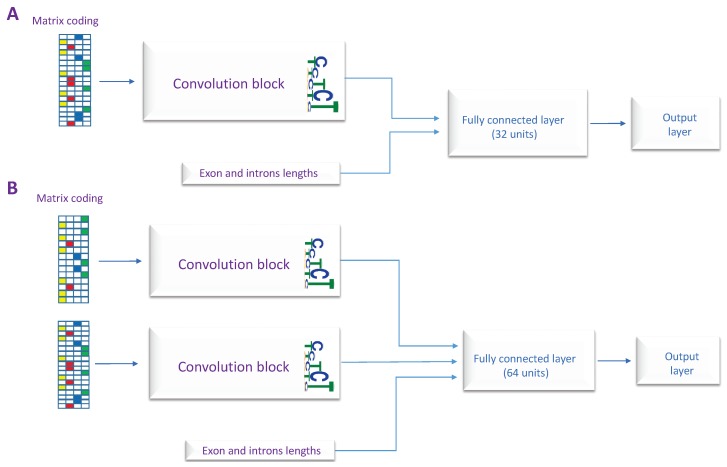
The architectures of the proposed models, (**A**) The first architecture uses single RNA sequence as input. (**B**) The second architecture takes two RNA sequences as inputs.

In this section, we explain our method and the designed models. In order to distinguish between different types of human internal exons, we propose four classification models: constitutive exons vs. skipped exons (CON-ES), constitutive exons vs. exons with used alternative 3’ (CON-ALT3), constitutive exons vs. exons with used alternative 5’ (CON-ALT5) and a general model to classify all the exons types called deep splicing code (DSC). Each one of these models uses one of the two designed architectures explained in Figure 3. The main component of the proposed architectures is a convolution neural network (CNN) that have gained significant attention in genomics application and predicting regulatory sequences [18,19]. While the first architecture has only one CNN block and takes as input only one RNA sequence, the second architecture uses two CNN blocks and two RNA sequences as inputs. The drawback of using the second architecture is the repetitive sequences in both inputs for the exons shorter than 140. However, as the sequences of the upstream and the downstream introns are different, each block learns to extract different features from the other. We use the features describing the lengths of the exon and neighboring introns in all the proposed architectures.

For the purpose of this study and depending on the classification type, the most suitable architecture is used along with the appropriate RNA sequences as input, i.e., when comparing skipped exons and constitutive exons, the RNA sequences from both the upstream and downstream exon junctions are considered, therefore the second architecture is used for this task. In contrast, to compare between constitutive exons and exons with alternative 3’ or 5’ splice site, only the RNA sequence from the upstream or the downstream exon junction, respectively, is considered. As we are interested in analyzing the local characteristics of the splice site itself. Consequently, we use the first architecture for these two classification tasks. The full list of the models and their corresponding architectures, inputs, and outputs are given in Table 2.

**Figure 4:**
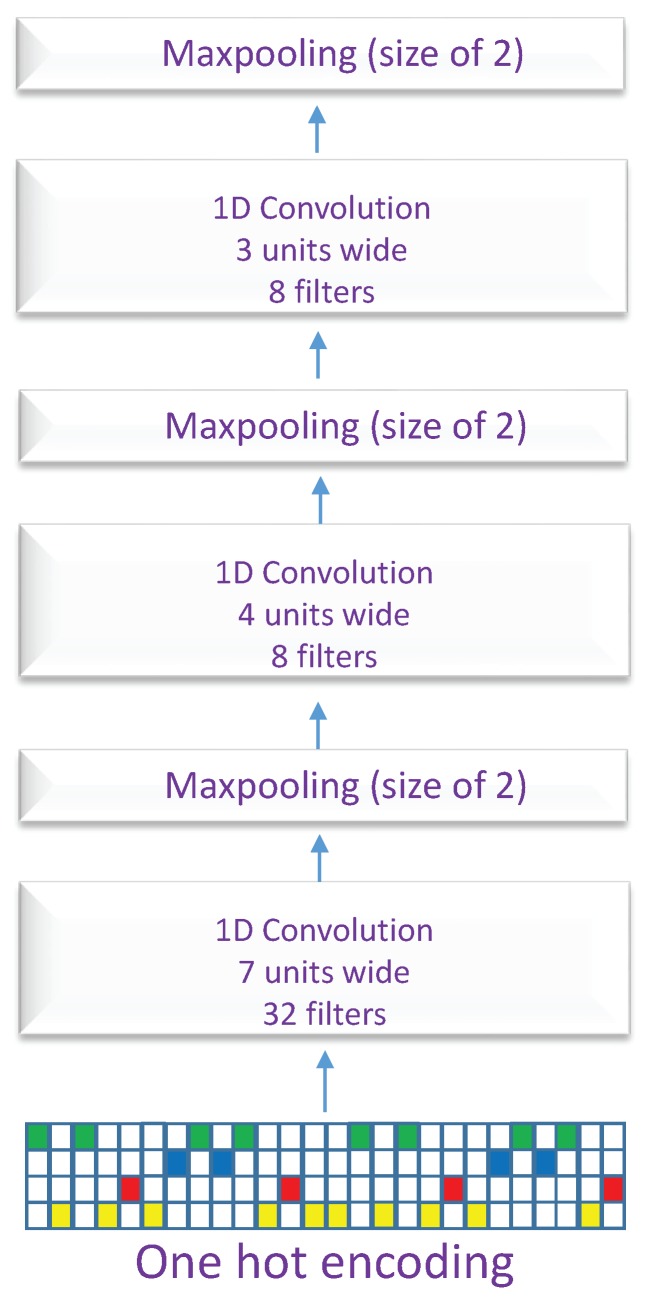
The detailed architecture of the convolution block.

Figure 4 shows the CNN block used in the two architectures. We used three convolution layers in every block. The first convolution layer has 32 filters with a window size of 7 units, the second convolution layer has 8 filters with a window size of 4 units, and the last convolution layer has 8 filters with a window size of 3 units. All convolution layers are followed by an activation layer ReLU, a dropout layer with a probability of 0.2 for reducing overfitting [35], and a max-pooling layer with window size and a stride step of 2 units. To find the optimal parameters of the CNNs, we perform an initial hyper-parameter selection using a grid search. Unlike machine learning techniques that require handcrafting the features before passing them to a classifier such as SVM, the CNN blocks take as input the raw DNA sequences and are trained in a supervised fashion to automatically extract the useful features for the classification task. The final CNN feature maps are flattened and concatenated together with the exons and introns lengths vector. All the combined features form the input of the fully connected layer which is followed by the final output layer. More specifically, CON-ALT3 and CON-ALT5 models have a fully connected layer with 32 nodes and followed by and an activation layer ReLU and a dropout layer with probability of 0.5, while the CON-ES and DSC models have a fully connected layer with 64 nodes and followed by and an activation layer ReLU and a dropout layer with probability of 0.5. Furthermore, the models, CON-ES, CON-ALT3, and CON-ALT5 perform binary classification with Sigmoid as an output layer while the last model DSC, which is a multi-class classification, uses a Softmax as an output layer.

### 2.4. Implementation Details

For the implementation, we use Python and the Keras library 1.3 with Tensorflow 1.0 [36] backend to construct the models. We use NVIDIA Titan XP GPU for accelerating the training process. All weights are initialized randomly by Gaussian distributions and all biases are initially set to 0. Learning is performed with Adam optimizer algorithm [37,38] with a learning rate of 0.0005 and a minibatch size of 100. Busch and Hertel [10] suggest splitting the training dataset into two subsets based on the exon’s splice usage to improve the discrimination power of the SVM classifier. The first subset contains the exons with high splice sites usage (>80% inclusion/usage) while the second one contains exons with low splice sites usage (<20% inclusion/usage). This setup results in training two different sets of classifiers for the two subsets. We follow a different design that aims to make a general model for all inclusion levels and splice site levels by using all alternative splicing events with various rates of occurrences. However, we focus on studying the effect of these frequencies on the accuracy of our model at the testing time.

As a quality control, we use 10-fold cross-validation during the training process. The dataset is randomly split into 10 folds. Eight folds are used for training, one for early stopping and the remaining one for testing. Previous studies have shown that it is hard to distinguish between constitutive exons and exons with high inclusion or with high usages of 3’ and 5’ [10,12]. Therefore, we split the testing set into two subcategories of exons with different inclusion or splice site usage levels. The first subset of events with inclusion/usage levels more than 80% is denoted as HEvents (high inclusion/usage levels). The second subset of events with the rest of events is denoted as MREvents (moderate to rare inclusion/usage levels). These cutoffs were based on the previous work [10] and our observation as explained in the next section. Table 3 presents the statistics of the two subsets. Using the cross-validation method, every one of the models: CON-ES, CON-ALT3, CON-ALT5, DSC is trained 10 times, a total of 40 models. We compute the average performance of the 10-fold with standard errors.

## 3. Results and Comparisons

The models are evaluated using AUC for each classification type. To inspect the effect of the inclusion/usage levels on the performance of the splicing code. We compute the prediction performance for all events in the testing set along with the two subsets explained in the previous section, i.e., MREvents and HEvents. The last subset (HEvents) includes exons with high inclusion/usage levels, i.e., skipped exons with very high inclusion behave very much like, and sometimes considered as, constitutive exons [39]. Accordingly, distinguishing between these events and constitutive exons is more challenging. Table 4 summarizes the obtained results from the 10-fold cross-validation on those subsets.

**Figure 5:**
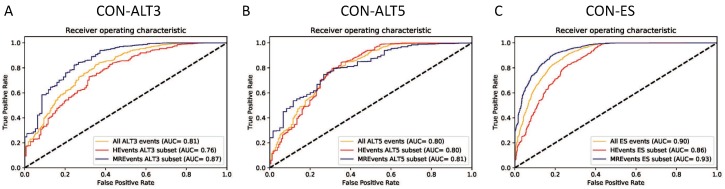
The performance of the proposed models in different usage levels, the curves for classification constitutive exons vs. (**A**) exons with alternative 3’ splice site for different usage levels (ALT3), (**B**) exons with alternative 5’ splice site for different usage levels (ALT5), (**C**) exon skipping events (ES) or cassette exons with different inclusion levels.

Figure 5A–C illustrate the plots of the true positive rate (TPR) against the false positive rate (FPR) for different thresholds (ROC curves). All three binary classifiers (CON-ALT3, CON-ALT5, and CON-ES) show higher performance on the MREvent subset than the HEvents subset. Similar patterns of the influence of the inclusion/usage levels are observed in all the 10 testing folds. They also achieve an average AUC of 0.81, 0.82, 0.90 for CON-ALT3, CON-ALT5 and CON-ES models, respectively, on all events of the dataset.

**Figure 6:**
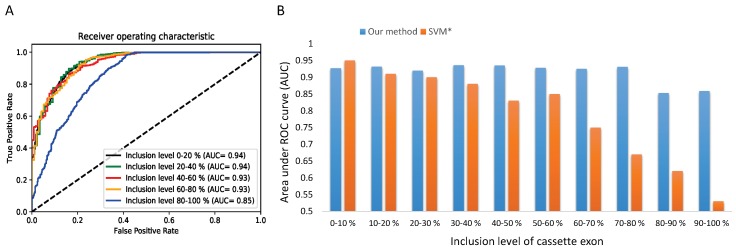
The performance of the CON-ES model in different usage levels, (**A**) ROC curves across 5 subsets of different inclusion levels generated from the first fold of the 10-fold cross-validation with similar observed patterns of the influence of the inclusion level in all the 10 testing folds. (**B**) A comparison of CON-ES model with an SVM classifier across 10 subsets of different inclusion levels. The AUC results are computed as the average of 10-fold cross-validation. The * symbol is Busch and Hertel [10].

To gain a deeper understanding on the performance of our method across various AS frequencies, we decide to go further in our analysis by focusing on the exons skipping category which is the most studied type of AS. We split the test set of cassette exons into 5 and 10 subsets according to the inclusion level. We compare the performance of the CON-ES model by computing the AUC for every inclusion interval. Figure 6A,B show the results across 5 and 10 subsets respectively. We observe that our splicing code distinguishes with very high and stable accuracy (0.92∼0.93 in AUC) the constitutive exons from the alternative spliced exons of rare to moderate inclusion. Furthermore, we notice a significant drop of the accuracy by around 0.08 for the subsets of the exons with inclusion level higher than 0.80 (HEvents subset). The obtained results confirm the finding of the previous method [10]. However, as shown in Figure 6B, the proposed method shows a lower gap difference and higher stability in the performance across the subsets than the previous work [10]. While both approaches show similar performance for the first two subsets of exons with very low inclusion level, the SVM accuracy drops dramatically to around 0.50 for the last subsets. These results demonstrate the efficacy of our method comparing to the use of the handcrafted RNA features that were unable to accurately distinguish the constitutive exons from the alternative exons with high inclusion level.

**Figure 7:**
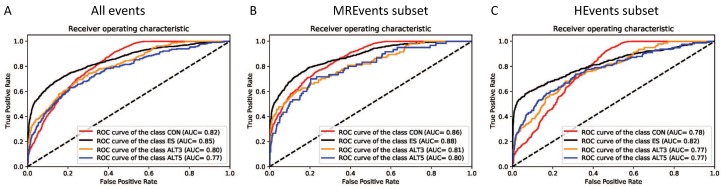
The performance of deep splicing code (DSC): The ROC curves of the DSC model for classification of different types of alternative splicing events, (**A**) All inclusion/usage levels, (**B**) MREvents subset: the event with inclusion/usage levels lower than 80%, and (**C**) HEvents subset: the events with inclusion/usage levels higher than 80%. All ROC curves are generated from the first fold of the 10-fold cross-validation with similar observed patterns of the influence of the inclusion/usage levels in all the 10 testing folds. Abbreviations of the classes are used as specified in Section “Dataset Preparation”.

Deep Splicing Code is a general model for classifying the human internal exons according to their alternative splicing behavior based on the RNA sequences, the exon length, and the neighboring introns lengths. DSC achieves an AUC of 0.83, 0.85, 0.81 and 0.77 for the classes CON, ES, ALT3, and ALT5, respectively. With an average AUC of 0.82. Likewise, when comparing on the MREvents and HEvents subsets as shown in Figure 7 and Table 5, we observed a similar drop of AUC by around 0.04 for the HEvents subset compared to MREvents subset. Another interesting observation is the relatively higher accuracy for the two classes CON and ES than the classes ALT3 and ALT5. The reason of this differences is the availability of the training samples for CON and ES classes in the human genome compared to the ALT3 and ALT5 classes as the performance of the deep learning algorithms depend greatly on the amount of the training data.

**Figure 8:**
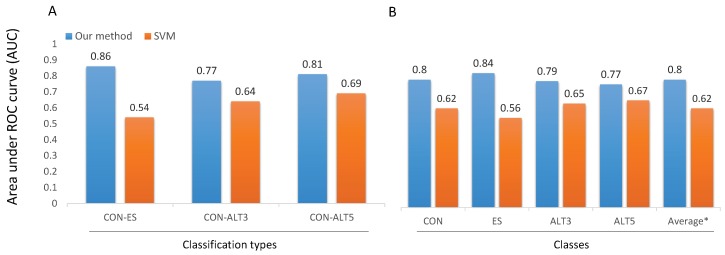
A comparison of the four models with SVM based method [10]. (**A**) Comparison of the three models: CON-ES, CON-ALT3 and CON-ALT5 with the SVMs binary classifiers. (**B**) General classification: comparison between DSC and a multi-classifier SVM. The performance is computed on the HEvents subset and measured as the average AUC. The * symbol is the average of the four classes.

Finally, in Figure 8 we compare the performance of the proposed models with the performance of SVM based one [10] on the challenging subset HEvents. In the binary classification (Figure 8A), all the three models (CON-ES, CON-ALT3, and CON-ALT5) largely outperform the SVM based one with an absolute improvement of around (0.12∼0.32) in term of AUC. In more details, CON-ES improves the AUC by 0.32, CON-ALT3 improves the AUC by 0.13, and CON-ALT5 improves the AUC by 0.12. On the other hand, in the multi-class classifier (Figure 8B), DSC model outperforms multi-classifier SVMs in all classes with an improvement of 0.18 of the average performance in term of AUC. More specifically, DSC improves AUC for CON, ES, ALT3, and ALT5 by 0.18, 0.28, 0.14, and 0.1, respectively. These observations reveal that the difficulty of the *in-silico* prediction of alternative splicing events depends highly on the frequency of occurrence of those events.

## 4. Models Interpretation and Discussion

These results demonstrated that CNNs were able to accurately predict the alternative splicing behavior of the exons using only the local sequence context of the exon itself and the exon/intron architecture. Remarkably, none of the features describing the donor site or the potential competitive splice-sites were used in our method such as the number of found putative splice sites and their strengths.

As a first step to gain insights into the performance of the models, we trained the Deep Splicing Code without the exon/introns lengths features and using the same parameters and dataset. We then observed a significant drop in performance in all classes. However, the average AUC was still 0.02 better than the SVM method [10] on the HEvents subset. The lowest decrease was recorded for the events with an alternative 3’ splice site and the highest one for the class of the cassette exons. These results confirmed the well-known effect of the exons and introns architecture on the regulation of AS, particularly on the cassette exon events as the probability of the exon to be skipped is higher for the ones flanked by long introns [15,34].

In addition to the exon/introns architecture factor, studies have shown that the AS behavior of an exon is primarily dictated by its immediate sequence context [10,12,14]. In fact, the general behavior of a constitutive exon across all types of tissues has been proven to be mainly driven by its splice-sites identities and the existence of binding motifs for splicing enhancers. Accordingly, the previous method included features such as the local secondary structures, phylogenetic conservation, binding sites for splicing regulators and splice-site strength which is estimated using MaxEntScan [40] etc. However, it was not a surprise that our method outperformed them as our prediction was based on 140 nt instead of the estimation of the splice sites strengths obtained from MaxEntScan [40] approach that was based only on 20nt. Moreover, the phylogenetic conservation value around the exon junction has also been shown to be an important feature [16,17,41]. Nevertheless, the conservation itself as a feature is not a recognized regulatory factor by the spliceosome. Several studies have suggested that conservation of sequences in that region can indicate the existence of other cis-acting elements that we are not yet aware of [17]. Instead of this, we accurately identified alternative splicing events using only raw RNA sequences without including any external information.

Despite the interesting findings, the drawback of using deep learning algorithms is the difficulty of interpreting the models. Neural networks have been criticized and considered as “black box” because the exact reason for their performance is not always clear. For the CNNs, One possible way is to visualize the learned filters as a similar style of the position weight matrices. Therefore, we extracted the learned kernels from the first layers of our models and converted them to motifs following the procedure of Quang and Xie [21]. Using TOMTOM tool [42], we matched the obtained motifs with the RNAcompete database [43] and SPLICE motif dataset [44] that contains matrix profiles of human canonical and non-canonical splice sites.

**Figure 9:**
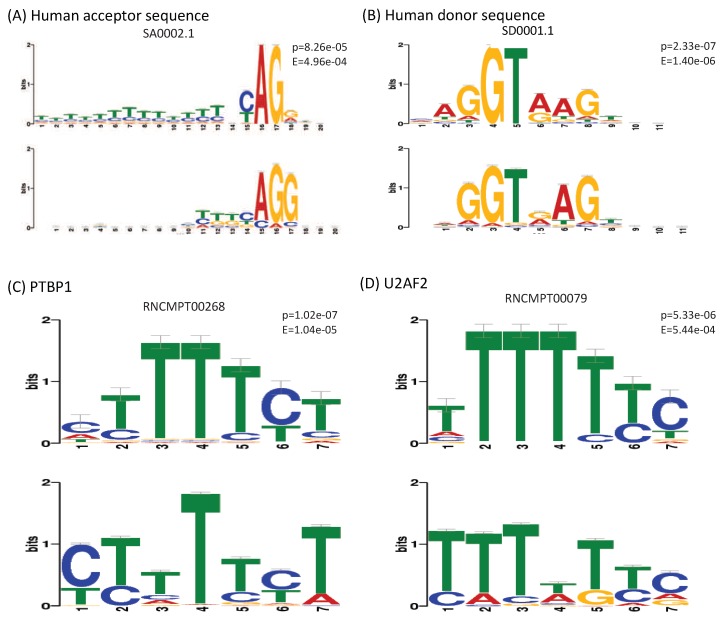
Visualization of the learned motifs extracted from the models: (**A**) CON-ALT3, (**B**) CON-ALT5, (**C**,**D**) CON-ES. The P and E values are obtained from TOMTOM [42]. First row represents the motifs from the RNAcompete database [43] while the second row represent the learned motifs by our models.

Figure 9 shows the highest certainty matches of the learned motifs against known ones. Particularly, we noticed that the model CON-ALT3 learned the motif of the canonical human acceptor sequence (Figure 9A), despite that all the samples used for training this model are all centered on AG sequences (both the positive and the negative samples). This observation reveals that this specific motif learned to search for potential alternative 3’ splice sites around the recorded splice site. Similarly, we found that a motif from the CON-ALT5 model matched with a high-certainty the canonical human donor sequence (Figure 9B). Which confirmed our initial hypothesis. Interestingly, although the previous method included the number of potential or putative splice sites along with their strength as features, our method was more accurate in finding the true alternative splice-site near the exon junctions. Which, again, approve the effectiveness of the deep learning representation.

In addition to the motifs of consensus splice sites, we found an important number of matches against the motifs of RNA-binding elements from the RNAcompete database. The strongest matches were the motifs extracted from the CON-ES model that almost perfectly match the ones of following splicing factors:

- PTBP1 (p = 1.02 × 10${}^{-7}$) (Figure 9C).
- U2AF2 (p = 5.33 × 10${}^{-6}$) (Figure 9D).
- PCBP2 (p = 1.25 × 10${}^{-6}$).
- TIA1 (p = 5.80 × 10${}^{-5}$).

All these proteins are known to be involved in pre-mRNA splicing or/and the regulation of AS events [45,46,47,48]. In addition, a recent study has shown that proteins such as PTBP1 and TIA group participate in the co-regulation of alternative exons by enhancing or silencing exons [49]. Our results confirm this finding. Furthermore, Deep Splicing Code was able to learn these motifs without pre-knowledge or explicit engineering. However, this is not the only reason behind the high performance of our method considering that the role of RNA binding proteins was known and their motifs were already used as features in the previous methods. Two main reasons may explain the success of DSC in classifying AS events. The first reason is that CNNs were able to extract motifs and features that we were not aware of besides the known ones. The second one lies in the nature of the deep learning algorithm itself. While the first layers of the network operate directly on the sequence and learn to scan for motifs of proteins, deeper layers learn the cooperative regulation of these motifs. This explains the success of using deeper and deeper networks in recent studies for modeling AS [50]. What’s more, these results open new opportunities to study and analyze the cooperative regulation of the RNA binding proteins in the AS using deep learning, which will be the direction of our future work.

To summarize, using the motif visualization technique, we observe that the trained CNNs learned to search for competitive alternative splice sites in addition to motifs of important splicing factors.

## 5. Conclusions

In this work, we developed a method for classification of the human internal exons according to their alternative splicing behavior using the local RNA sequences only. Firstly, we generated a dataset of AS events for training and evaluating the proposed computational models. We described the proposed architectures based primary on the deep CNN to automatically learn the features from the sequences. To demonstrate the effectiveness of this approach, we designed four models to distinguish between constitutive exons and various exons with alternative splice sites. We showed that all our models outperformed the existing method by a large margin. Furthermore, The Deep Splicing Code is a generalization of our method for classifying all AS types from sequence and without any engineered features or domain knowledge. When evaluating our models’ performance, we observed that the accuracy of the classification drops for the highly included cassette exons and exons with high usage of the alternative 3’ and 5’ splice sites. These observations endorse the complexity of the regulation of alternative splicing in the human genome. However, the results reveal that even high included exons or exons with highly used alternative splice site have distinguishable local characteristics from constitutive exons. Future works include analyzing deeper layers for a better understanding of co-operative regulation of RNA proteins and predicting novel AS events due to mutations. Finally, a free-access web-server was developed for the proposed method at https://home.jbnu.ac.kr/NSCL/dsc.htm.

## Figures and Tables

**Table 1:** Dataset statistics.

AS Type	Cons	ES	Alt3	Alt5
**Number of events**	39,128	11,590	2332	2172

**Table 2:** The proposed models and their corresponding architectures, inputs, and outputs.

The Model	Architecture Used	Inputs	The Output Layer
CON-ES	Architecture B	Upstream and downstream sequences	Sigmoid
CON-ALT3	Architecture A	Upstream sequence	Sigmoid
CON-ALT5	Architecture A	Downstream sequence	Sigmoid
DSC	Architecture B	Upstream and downstream sequences	Softmax

**Table 3:** Statistics of the two subset categories HEevents and MREvents.

AS Type	HEevents	MREvents
**ES**	4952	6638
**ALT3**	1388	944
**ALT5**	1568	604

**Table 4:** The performance of the proposed models measured by the AUC for all events and the two subsets with standard errors given in parentheses.

Model	All Events	MREvents Subset	HEvents Subset
CON-ES	0.899 (±0.008)	0.928 (±0.007)	0.858 (±0.015)
CON-ALT3	0.812 (±0.016)	0.870 (±0.016)	0.772 (±0.022)
CON-ALT5	0.821 (±0.014)	0.838 (±0.028)	0.813 (±0.017)

**Table 5:** The performance of deep splicing code (DSC) measured by the AUC for all events and the two subsets with standard errors given in parentheses.

Exon Type	All Events	MREvents Subset	HEvents Subset
Constitutive exons	0.835 (±0.007)	0.870 (±0.005)	0.798 (±0.001)
Cassette exon	0.858 (±0.007)	0.878 (±0.008)	0.844 (±0.012)
Exon with an alternative 3${}^{\prime }$-splice site	0.819 (±0.014)	0.832 (±0.025)	0.795 (±0.020)
Exon with an alternative 5’-splice site	0.772 (±0.017)	0.788 (±0.029)	0.774 (± 0020)
Average of the four classes	0.821	0.842	0.803

## Sample Availability

The datasets generated for this study and source code are freely available at https://home.jbnu.ac.kr/NSCL/dsc.htm.
